# Combined *in situ* Physical and *ex-situ* Biochemical Approaches to Investigate *in vitro* Deconstruction of Destarched Wheat Bran by Enzymes Cocktail Used in Animal Nutrition

**DOI:** 10.3389/fbioe.2019.00158

**Published:** 2019-06-26

**Authors:** Marine Deshors, Olivier Guais, Virginie Neugnot-Roux, Xavier Cameleyre, Luc Fillaudeau, Jean Marie Francois

**Affiliations:** ^1^LISBP, UMR INSA-CNRS 5504 & INRA 792, Toulouse, France; ^2^Cinabio-Adisseo France S.A.S., Toulouse, France; ^3^Fédération de Recherche FERMAT (Fluides, Energie, Réacteurs, Matériaux et Transferts), Université de Toulouse, CNRS, INPT, INSA, UPS, Toulouse, France

**Keywords:** wheat bran, enzymes cocktail, viscocity, fragmentation, solubilization

## Abstract

Wheat bran is a foodstuff containing more than 40% of non-starch polysaccharides (NSPs) that are hardly digestible by monogastric animals. Therefore, cocktails enriched of hydrolytic enzymes (termed NSPases) are commonly provided as feed additives in animal nutrition. However, how these enzymes cocktails contribute to NSPs deconstruction remains largely unknown. This question was addressed by employing an original methodology that makes use of a multi-instrumented bioreactor that allows to dynamically monitor enzymes in action and to extract *in-situ* physical and *ex-situ* biochemical data from this monitoring. We report here that the deconstruction of destarched wheat bran by an industrial enzymes cocktail termed Rovabio® was entailed by two concurrent events: a particles fragmentation that caused in <2 h a 70% drop of the suspension viscosity and a solubilization that released <30 % of the wheat bran NSPs. Upon longer exposure, the fragmentation of particles continued at a very slow rate without any further solubilization. Contrary to this cocktail, xylanase C alone caused a moderate 25% drop of viscosity and a very weak fragmentation. However, the amount of xylose and arabinose from solubilized sugars after 6 h treatment with this enzyme was similar to that obtained after 2 h with Rovabio®. Altogether, this multi-scale analysis supported the synergistic action of enzymes mixture to readily solubilize complex polysaccharides, and revealed that in spite of the richness and diversity of hydrolytic enzymes in the cocktail, the deconstruction of NSPs in wheat bran was largely incomplete.

## Introduction

Wheat is a major energy source in broilers feed worldwide due to its high content of storage polysaccharides (i.e., starch; Amerah, 2015). However, cereals also contain 12–15 % of non-starch polysaccharides (NSPs); (Knudsen, 2014; Onipe et al., 2015), which are mainly composed of arabinoxylans (AXs), cellulose and β-glucans (Maes and Delcour, 2002). Wheat bran AXs, which amounts to about 70% of the total NSPs, are linear chains of β-(1,4)-linked D-xylose on which α-L-arabinofuranosyl units are attached as single units on carbon 2 and /or carbon 3 of the xylosyl unit of linear chains (Ordaz-Ortiz and Saulnier, 2005; Knudsen, 2014). NSPs are recognized as anti-nutritional factors in animal nutrition because (a) they may trap nutrients (i.e., starch and proteins) within their fibrous structure, (b) they can act as chelators of minerals such as Ca^2+^ or Fe^2+^, and (c) they can reduce nutrients adsorption due to their high molecular weight that enhances the viscosity in the digestive tract ion (Ravindran, 2013; Lafond et al., 2015). Therefore, since the 80th the common practice in animal nutrition has been to complement animal diets with enzymes cocktails (Bedford and Partrige, 2011). This practice has shown significant beneficial zootechnic effects, such as improving animal performance and reducing excreta by a better nutrient absorption, resulting in a positive environmental impact of animal farming (Fuente et al., 1995; Gunawardana et al., 2010; Lafond et al., 2011a; Lei et al., 2016). While these beneficial effects are assumed to be due to the assimilation of NPSs, there is yet no direct experimental data, at least in the field of feed nutrition, showing how these NSP substrates are deconstructed and solubilized by these enzymes cocktails. In addition, there is a need to unravel the mode of action of these cocktails on such complex polysaccharides to eventually improve their efficiency in animal nutrition.

Although enzymatic deconstruction of biomass is largely investigated in the field of biorefinery and biofuels (Himmel et al., 2007; Dodd and Cann, 2009; Kubicek and Kubicek, 2016), in feed nutrition, such studies are restricted to global effects on nutrient digestibility or nutrients intake (Aulrich and Flacoxwsky, 2001; Malathi and Devegowda, 2001; Vahjen et al., 2005; Brufau, 2006; Amerah, 2015; Lafond et al., 2015). Due to the heterogeneity and insolubility of wheat bran, unraveling the deconstruction of insoluble NSPs by NSPases is actually challenging, as it requires both physical tools to observe and monitor particles deconstruction and biochemical methods to identify and quantify the solubilized matter during the treatment. This challenge has been therefore addressed by a multiscale approach which combines *in-situ* physical (i.e., rheological, morpho and granulometric data) and *ex-situ* biochemical (i.e., solubilization and nature of the products solubilized) analyses, by mean of a multi-instrumented bioreactor. This multiscale approach has been previously applied to investigate deconstruction of lignocellulosic-based biomass for biorefinery purposes (Nguyen et al., 2013; Le et al., 2017). In addition, this methodological approach was very appropriate for our study since at variance of several works employing mixture of pure enzymes with known catalytic activities (Rosgaard et al., 2007; Vaaje-Kolstad et al., 2010; Goldbeck et al., 2014; Frommhagen et al., 2017), here we used an industrial cocktail termed Robavio®. Although the protein content of this cocktail has been previously characterized by a proteomic study (Guais et al., 2008, and see Figure S1), only a few of the 20 different glycosyl hydrolases listed in this cocktail have been characterized so far (Guais et al., 2010; Lafond et al., 2011b, 2014; De La Mare et al., 2013). To further illustrate the compliance of this methodology to investigate the synergistic action of enzymes mixture in the deconstruction of biomass, we compared the action of xylanase C, which is the most active glycoside hydrolase in Rovabio® (Lafond et al., 2014), to that of this cocktail in the deconstruction of wheat bran. Altogether, our multiscale analysis unraveled that the deconstruction of the wheat bran NPSs by Rovabio® occurred by a concurrent particles fragmentation and solubilization, which unexpectedly was limited to <30% of the initial wheat bran NSPs.

## Materials and Methods

### Substrates and Enzymes

Wheat bran was obtained from “La Minoterie de la Save” (Grenade sur Garonne, France) and was extensively destarched by lixiviation according to Raynal-Ioualalen (1996). Briefly, one batch (~3.5 kg) of wheat bran was suspended in water at 10% vol/vol. Four consecutive washes were applied in a stirred tank (*V* = 40 L) at 350 RPM and 40°C during 15 min for the first one and 10 min for the following ones. Between each step, suspension was clarified by decantation and the supernatant containing solubilized starch (called “starch milk”) was removed. Then fresh water was added to maintain a constant solid/liquid ratio at 10%. Wheat bran was finally rinsed by percolation suing bag filter with a cut-off of 50 μm, dried with incoming compressed air and stored at −18°C. The amount of NSPs and residual starch in this material was estimated, respectively, to 71% and to <5% of the wheat bran after acid hydrolysis.

Rovabio® (abbreviation of Rovabio® *Excel*) is an enzymatic cocktail obtained from the secretome of *Talaromyces versatilis* fungus and commercialized by Adisseo SAS (Commentry, France, http://feedsolutions.adisseo.com/en/). The enzyme composition of this cocktail has been determined by mass spectrometry (Guais et al., 2008; see Figure S1) and has been classified according to main CAZy categories (http://www.cazy.org/). As indicated in the supplementary data (Figure S1), it contains several types of glycoside hydrolases herewith termed NSPases including endo-β-xylanases, β-glucanases, pectinases, cellulases, and L-α-arabinofuranosidases. Due to the vast diversity of hydrolytic activities, the global hydrolytic capacity of this cocktail is expressed in viscosity units per ml of suspension (U_xylanase visco_/mL) using wheat arabinoxylans (P-WAXYH purchased from Megazyme) as the substrate. This activity corresponds to the fluidity reduction of one unity (dimensionless) per minute under the analysis conditions (pH 4.0, 0.1 M Na acetate buffer, 41°C). The total activity of Rovabio® corresponds to 24,000 U_xylanase visco_/mL. The “global” enzymatic activity of endo-1,4-β-xylanase in the cocktail can be also determined by the 3,5-dinitrosalicylic acid (DNS) colorimetric method (Mckee, 2017) using a solution of 1.5% of birchwood xylan as substrate in 75 mM of NaAcetate buffer pH 4.0. The ratio between viscosity units and xylanase activity is around 9 (V. Neugnot-Roux, unpublished data).

Production of recombinant endo-xylanase C encoded by *T versatilis XynC* (Lafond et al., 2014) has been subcontracted to GTP biotech company (https://fr.gtptech.com/). Briefly, it consisted of a 50 L fermentation of *Pichia Pastoris* strain GS115 that expressed *T. versatilis XynC* cloned in a pPIC9K plasmid under the strong *AOX1* promoter and bearing a secretory sequence to release the protein in the supernatant. The enzyme showed to be pure by SDS page analysis and exhibited 30 000 U_xylanasevisco_/mL.

### Experimental Pilot Set-Up

**Figure 1:**
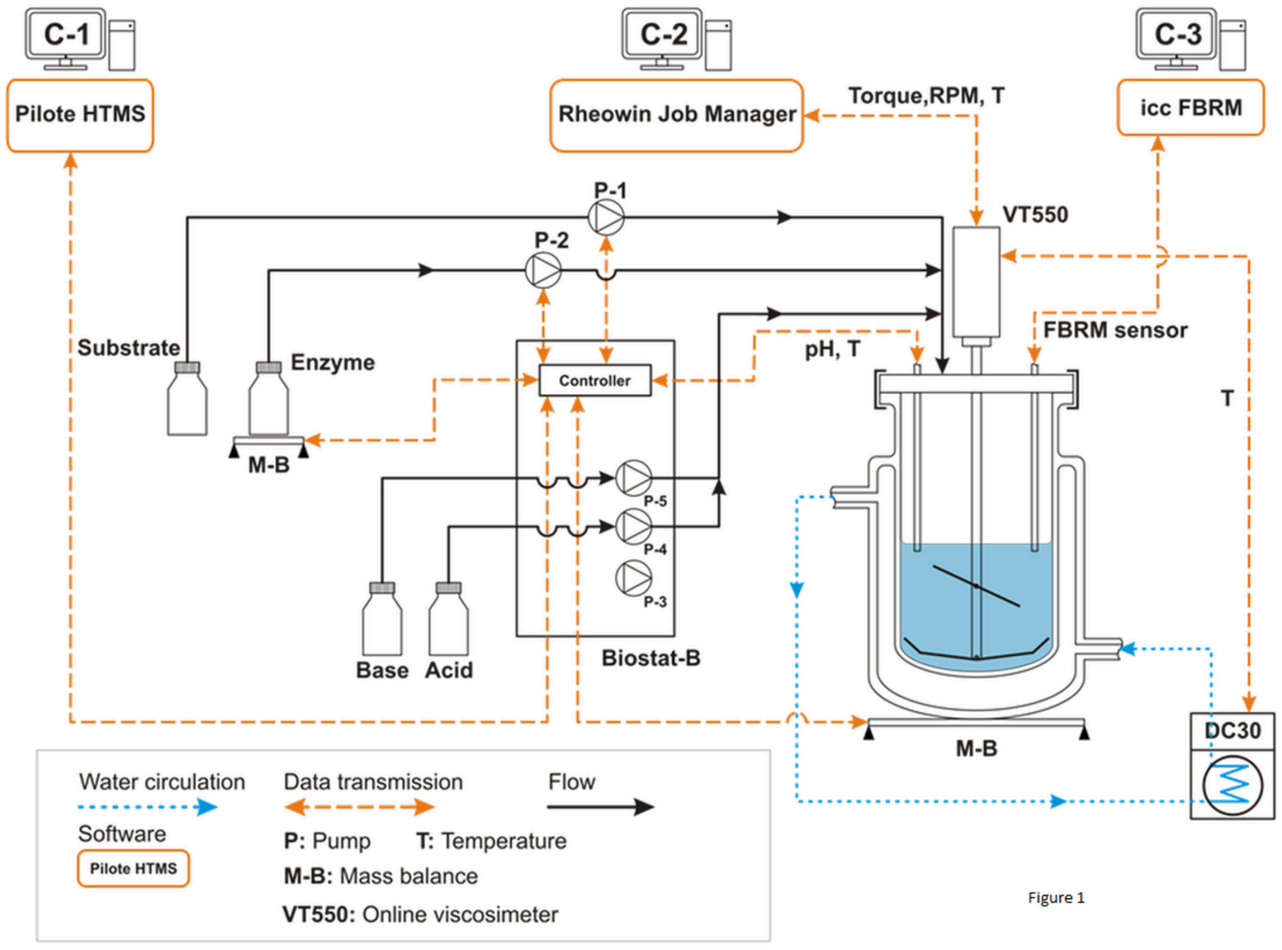
Process and instrumentation diagram of the experimental setup. All experiments were conducted in batch mode. Pump P1 (archimede screw pump) is used to load progressively the reactor with the wheat bran substrate in order to generate a homogeneous suspension under permanent mixing. Pump P2 (volumetric peristaltic pump) is used to add quickly the enzymatic cocktail at a defined time (From Le et al., 2017).

A general description of the experimental system is illustrated in Figure 1. It includes a double jacket glass bioreactor (*d* = 130 mm, *H* = 244 mm, *V* = 2.0 L) equipped with a home-designed impeller system associated with several *in-situ* sensors (temperature, pH, rotation speed, torque, FBRM). The impeller is composed by a three inclined blades located at 75 mm height from the bottom (diameter: 73.5 mm, angle: 45°, *h* = 38 mm) and a close bottom mixer including 2 large blades (diameter: 120 mm, *h* = 22 mm). A Haake VT550 viscometer (Thermo Fisher Scientific ref: 002-7026, 0.5-800 RPM ±0.1%, 100–30,000 μN.m ±0.5 %FSD) was used to ensure mixing at specific rotation speed as well as *in-situ* torque measurements. The temperature was controlled by water circulation (combined cryostat Haake DC30-K20, −50/+200°C ±0.01, Thermo Fisher Scientific) through the double jacket of the bioreactor. The viscometer and the cryostat were controlled by original software from Haake (RheoWin Job Manager) that also ensured real-time monitoring (temperature, torque, and mixing rate). The pH of the suspension was controlled and auto-adjusted by a Biostat-B (Sartorius Stedim Biotech). The bioreactor also holds a focused beam reflectance sensor (FBRM-G400-Mettler Toledo) to measure particle chord length (l_*c*_) and the number of particle counts (*Nc*) per second and size classes.

### Chemical and Biochemical Analysis

Samples (4 × 3 mL) were collected during hydrolysis experiments (6 time points per experiment, under continuous stirring tank to maintain suspension homogeneity) using a graduated pipette, in 15 mL falcon conical tubes placed in an ice-cold bath to readily stop the enzymatic activity. One half of the samples was centrifuged (10 min at 4,000 RPM) and stored at −18°C for biochemical analyses and the other half was directly stored at 4°C for physical analyses. To prevent any alteration of fiber properties, *ex-situ* physical analyses were performed within 24 h after sampling.

#### Determination of the Solubilization Rate

The solubilization rate corresponds to the soluble matter released in the supernatant during enzymatic treatment of destarched wheat bran. It was determined by drying 2 mL of supernatant at 105°C on Fontainebleau sand (Ref 310-127-6) until stability of the measured mass using a Moisture Analyzer (MA 100H Moisture analyzer, Sartorius, 30–180°C). These values were used to evaluate the residual dry matter concentration (Cm) remaining at different times during the treatment of insoluble wheat bran.

#### Biochemical Methods

Soluble oligosaccharides released in the supernatant during the treatment of wheat bran were subjected to sulfuric acid according protocol described elsewhere (Francois, 2006) in which the first step was skipped to hydrolyze them into their corresponding monosaccharides. After hydrolysis and neutralization with Ba(OH)_2_, samples were filtered through 0.22 μm filters and sugars were determined by High-Performance Anion Exchange Chromatography (HPAEC). The HPAEC was carried on a ICS 3000 system (Thermofisher Scientific, Courtaboeuf, France) using a CarboPac SA10 analytical column (250 by 4 mm) with a guard column CarboPac SA10, an isocratic elution of 1 mM NaOH at 35°C and a flow rate of 1.5 mL/min. To determine the monosaccharides that were directly released in the supernatant during the enzymatic treatment, a CarboPac PA1 analytical column (250 mm × 4 mm) with a CarboPac PA1 guard column, and an isocratic elution of 18 mM NaOH at 25°C with a flow rate of 1 ml/min were employed. The monosaccharides were converted to the equivalent polysaccharides using the conversion factor of 0.88 for pentose (arabinose and xylose) and 0.90 for hexose (mannose, galactose and glucose) (Sluiter et al., 2008), and the obtained values were multiplied by 1.1 to correct for the roughly 10% loss of monosaccharides during the acidic treatment in accordance with (Wijaya et al., 2014; Zhou and Runge, 2014) and our data (not shown).

Protein concentration was determined by the BCA (BiCinchoninic acid Assay) assay kit (Pierce Thermo Scientific, Illinois, USA) (Smith et al., 1985) using bovine serum albumin as standard.

### Physical Analysis

#### *In-situ* Viscosity Measurement

The *in-situ* viscosity was determined from real-time monitoring of torque and mixing rate. This measure requires to determine *Kp* and α which are two constants that only depend on the mixing system geometry and *Np*_0_ which is the mixing power number for turbulent flows. For Newtonian fluids and in laminar flows, the product of the mixing Reynolds number (*Re*) by the mixing power number (*Np*) is constant and written as:

(1) $$\begin{array}{r} Np\;=Kp/Re \end{array}$$

This power consumption curve *Np* = *f*(*Re*) was characterized using Newtonian reference fluids: distilled water, glycerol, and Marcol 52 oil (Exxon Mobil). Equation (1) for laminar regime can be extended to turbulent ones (until a critical *Re* value) and described by a unique equation: Equation (2).

(2) $$\begin{array}{r} Np={\left[{\left(\frac{Kp}{Re}\right)}^{\alpha }+Np_{0}^{\;\alpha \;}\right]}^{1/\alpha } \end{array}$$

Experimental results on our system gave *Np*_0_ = 0.17, α = 0.75, *Kp* = 115.2 and showed that a laminar regime prevailed up to *Re* = 41 (Nguyen et al., 2013; Le et al., 2017).

For a non-Newtonian fluid, Equation (2) is still valid as long as a generalized Reynolds number is used. It is calculated using the Metzner-Otto concept which introduces a constant *Ks* that only depends on the geometrical characteristics of the mixing system (Metzner and Otto, 1957). The *Ks* value is determined experimentally using 0.04–0.1% vol/vol shear-thinning xanthan solutions prepared in a saturated solution of glucose and sucrose as reference fluids and was found in our system to be equal to 32. The concept can be extended up to transition flow (Jahangiri et al., 2001). In this study, the application of a power consumption curve to calculate suspension viscosity and establish an *in-situ* rheogram was extended to transitional flow, which is equivalent to *Re* < 1,000. In turbulent flow, determination of viscosity is limited by the power consumption curve up to *Re* = 30,000 (above this value, *Np* is almost constant).

The Non-Newtonian behavior, described by the flow behavior index (n) was estimated from flowing curves (μ-N). It was determined every 15 min by adjusting mixing rates (mean shear rates) from 170 to 200 RPM for 1 min, and from 200 to 150 RPM for 1 min. Data acquisition period was adjusted to 20 s at 170 rpm and reduced to 10 s at 150 and 200 rpm. During both steps, mean torque was determined after stabilization.

#### *Ex-situ* Laser Light Diffraction (LLD)

The volume-weighted particle size distribution (PSD) was determined by Laser Light Diffraction (LLD, Mastersizer 2000, Malvern Inst., range from 0.02 to 2,000 μm, red λ = 632.8 nm and blue λ = 470.0 nm light) using Mie scattering theory. A known volume of suspension (1–3 mL) was added to a close water circulation loop (20°C ± 2) in order to obtain laser obscuration between 5 and 40%. The whole suspension was mixed by a Heidolph magnetic stirrer at 200 min^−1^ with the circulation loop maintained by a Masterflex L/S model 7553-79 at a pump speed of 240 min^−1^. The measurements of each sample were performed in triplicate at three different dilution rate and the average data was taken. Laser diffraction analysis converts the intensity of scattered light into PSD value, which is assimilated to a diameter of equivalent sphere, d_se;_. Volume-weighted distribution, function of equivalent sphere-diameter, *E*_*v*_(*d*_*se*_) was multiplied by dry matter concentration (Cm) to take into account the loss of material due to solubilization during the enzymatic treatment.

#### *In-situ* Focus Beam Reflectance Measurement (FBRM)

Focus beam reflectance measurements (FBRM) enable *in-situ* quantification of small particles size < 100 μm through the estimation of the chord length (lc) and distribution of the chord length population (CLD). *In-situ* CLD of particles was analyzed using an FBRM® G400 probe (Mettler Toledo, range: 0.1–1,000 μm, laser light source λ = 795 nm, laser source rotation: 2 m/s). This probe was placed in the bioreactor with a 45° inclination to the liquid stream according to the Mettler-Toledo specification (https://www.mt.com/dam/product_organizations/autochem/fbrm2016/ParticleAnalysis_042816.pdf) in order to avoid any particle sticking and recirculation within the focus plane. The probe allowed for real-time tracking of chord length and particle count during enzymatic hydrolysis. Thousands of individual chord lengths are measured each second to produce the number chord length distribution (CLD), which is the fundamental measurement provided, by FBRM®. Consequently, the number-weighted CLD, *E*_*n*_(*l*_*c*_), and the average number of chord length counted per second, *N*_*c*_, are used as indicators to describe population.

## Results

### Set-Up of the Experimental System and Operational Conditions

The originality of our study was to use a multi-instrumented bioreactor (Figure 1) that allows to analyze at different scale, i.e., rheology, morphogranulometry and biochemistry, the deconstruction of insoluble biomass from various origins by hydrolytic enzymes either alone, or in a mixture under well-controlled operating conditions. In the present study, we determined the efficiency of the Rovabio®, a commercial enzymatic cocktail enriched with more than 20 different glycoside hydrolases (Guais et al., 2008) to deconstruct the non-starch polysaccharides (NSPs),which can amount to 46% of wheat bran (Knudsen, 2014). At first, we had to remove as much as starch as possible by lixiviation method since this polysaccharide was not of any interest in this study but more importantly, it interfered with our physical (i.e., viscocity) analysis. This treatment allowed us to work with a destarched material that contained <5% of starch, more than 70% of NSPs, 15% of proteins as well as 10% of uncharacterized matter (data not shown). To investigate the deconstruction of this material with enzymes alone or cocktail (Rovabio®), a multi-instrumented bioreactor (Figure 1) was set up which enables a dynamic monitoring at the macroscopic (rheology), microscopic (particle size and number) and biochemical scales (sugars and proteins solubilization) of the enzymatic deconstruction under well-controlled operating conditions.

As a starting point in this work, the critical concentration (C_*crit*_) that marked the transition from a dilute to a semi-dilute suspension was determined from flowing curves. This value was obtained by applying various mixing rates (from 10 to 250 RPM) to destarched wheat bran suspended in potassium phosphate buffer (pH 4, 41°C) at concentration ranging from 10 to 86 g/L. Moreover, operating conditions were defined to approximate those encountered in the poultry anterior digestive system, which corresponds to a high dry matter (>50 g/L), temperature of 41°C and pH around 4.0 (Svihus, 2014). In agreement with literature data (Geddes et al., 2010; Nguyen et al., 2015), the viscosity and the non-Newtonian behavior of the suspension were strongly dependent on substrate concentration. A C_*crit*_ value of 50 g/L was obtained and operational conditions were fixed at 1.5 C_*crit*_ to ensure a laminar regime during the time of the experiment. In addition, prior to enzymes addition in the bioreactor, the destarched wheat bran suspension was subjected to a thermal treatment. This implied to raise the temperature of the suspension in the reactor up to 85°C and maintained at it for 1 h to denature xylanase inhibitor proteins XIP (xylanase inhibitor protein) and TAXI (*Triticum aestivum* endoxylanase inhibitor) present in the wheat bran (Furniss et al., 2002; Juge, 2006). Upon return at 41°C and after stabilization of the suspension (stable viscosity value), enzymes (either Rovabio® or xylanase C) were added at an equivalent of 400 U_xylanase visco_ per gram of suspended dry matter. Samples were regularly taken over a 6 h period to monitor and quantify physical and biochemical parameters.

### Destarched Wheat Bran Deconstruction Monitored by *in-situ* Viscosity

**Figure 2:**
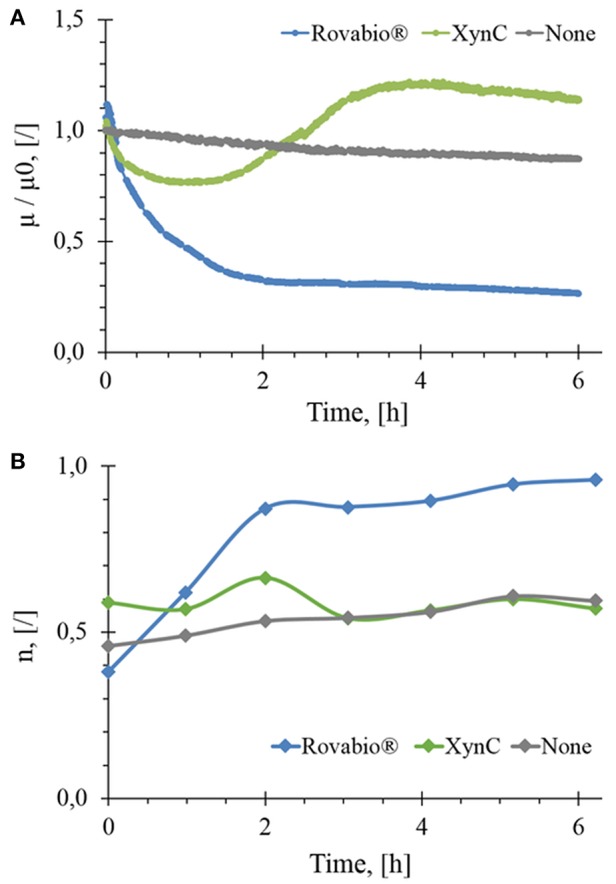
Normalized *in-situ* viscosity **(A)** and flow behavior index **(B)** as a function of the hydrolysis time of wheat bran suspension by Rovabio^®^ **(A)**, pure xylanase C (XynC) and without enzyme (None). Operationg conditions: destarched wheat bran suspended in a potassium phosphate 0,1M at 41°C, pH 4.0, initial concentration 74 g/L, Robavio Advance and xylanase C 400 U_xylanase visco_/g.

Destarched wheat bran suspended at about 74 g/L and mixed at 170 RPM gave an initial viscosity μ_0_ of 0.206 Pa.s after suspension homogenization and stabilization. In the absence of NSPases, a very weak linear decrease of viscosity (<10%) over 6 h was observed, which can be due to mechanical disaggregation of insoluble particles by the mixing system (Figure 2A).

Upon addition of Rovabio® (400 U_xylanase visco_/gdm), the viscosity dropped by 70% in the first 2 h following a 1st order decay, then slowed down in the next 4 h to stabilize at a value of 0.05 Pa.s after 6 h. To verify whether the slow reduction of viscosity could result from an inhibition of some enzymes in the cocktail by products released in the supernatant, fresh wheat bran was suspended in the supernatant of a 6 h-treated wheat bran suspension and the same dose of Rovabio® was then added to this new suspension. Since a similar viscosity drop as in the first experiment was recorded, we concluded that the slowing down in viscosity drop was not due to enzymes inhibition. Two additional experiments were carried out to confirm that the enzymes cocktail was not inactivated after 6 h treatment, as monitored by change in the viscosity. In the first experiment, a fresh Rovabio® solution in high excess of >1,000 U_visco_/gdm was added to the 6 h treated wheat bran suspension and no further decrease of viscosity was observed. For the second experiment, the insoluble fraction obtained after the 6 h treatment was collected and re-suspended in a fresh buffer solution. The viscosity of this latter suspension was not further modified upon addition of high excess of Rovabio® (data not shown).

We then investigated effects of pure xylanase C, which corresponds to the most active endo- β-1,4-xylanase present in Rovabio® (Lafond et al., 2014) on destarched wheat bran. As it can be seen in Figure 2A, the viscosity of the suspension decreased by only 25% during the first hour, followed by an overtake above the initial value between 2 and 4 h (+20%) to finally stabilized at a relative viscosity that was 14% above the initial value. Taking into account that the drop in viscosity is a direct physical indication of biomass deconstruction (Dunaway et al., 2010; Wiman et al., 2011; Nguyen et al., 2013), this result indicated that xylanase was much less efficient than Rovabio® likely because of the synergistic action of the various and different hydrolytic enzymes present in this cocktail.

The rheological behavior of the wheat bran suspension during the treatment with either Rovabio® or xylanase C was monitored by measuring the flow behavior index (*n*). These measurements were feasible since the destarched wheat bran suspension remained in laminar and transitory regimes (*Re* < 700). Figure 2B shows that the shear-thinning behavior of the suspension was changing upon treatment with the enzyme cocktail, as shown by an increase of the flow behavior index (*n*) from 0.4 to near 1 after 2 h of treatment, which indicated that the suspension has reached a Newtonian behavior. On the contrary, with the xylanases, the shear-thinning behavior of the suspension remained unchanged *(n* ~ 0.5), even though we noticed a decrease in viscosity by 25% within the first 2 h after addition of the enzyme (Figure 2A).

### Granulometric Analysis of Destarched Wheat Bran Deconstruction

**Figure 3:**
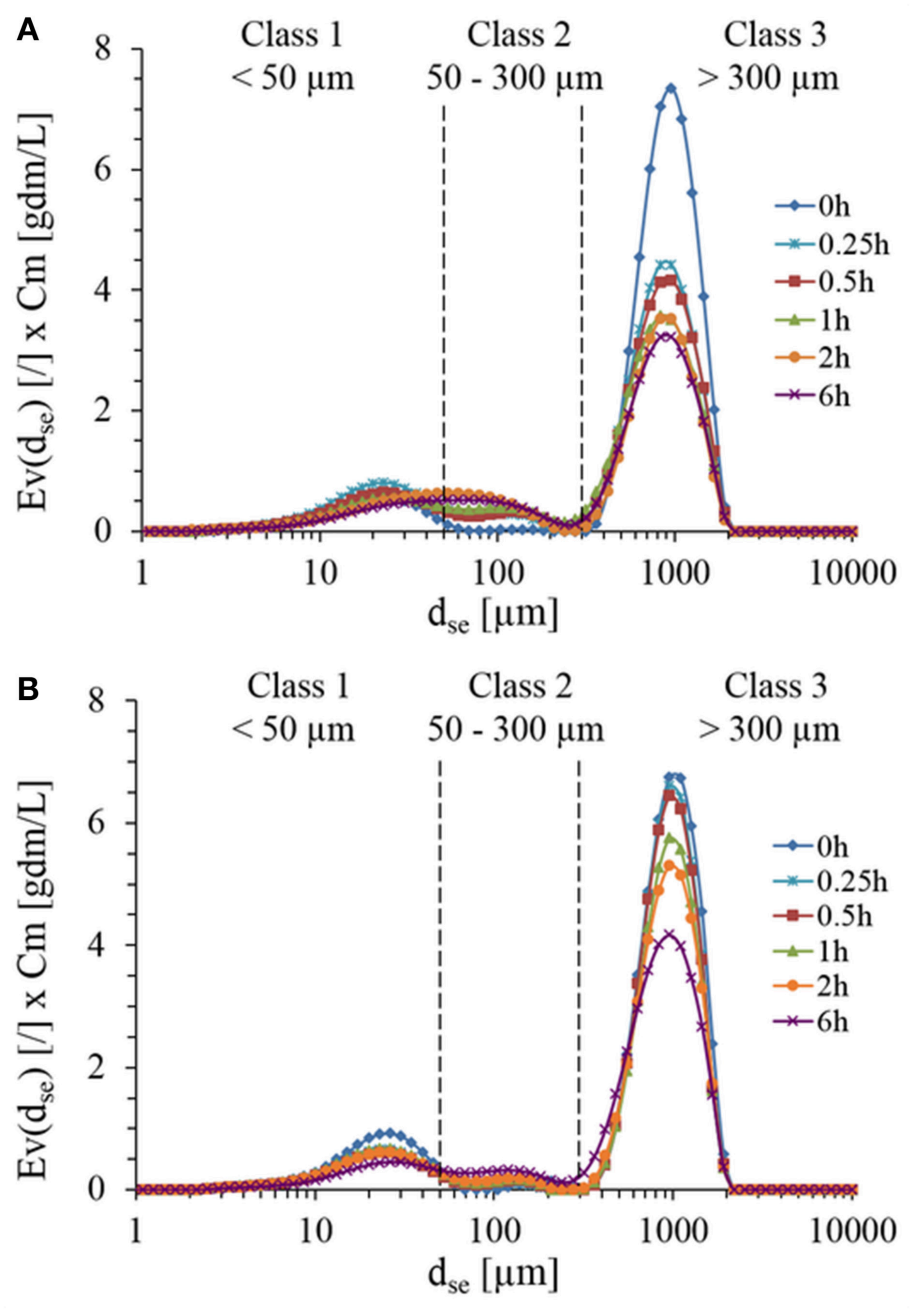
Particles size distribution during the treatment of destarched wheat bran suspension in the presence of Rovabio® **(A)** and xylanase C **(B)**. The particle size distribution was assimilated to a diameter of equivalent sphere (dse) and was plotted with respect to the abundance of particle size which was obtained by multiplying Ev by the concentration of wheat bran suspension (Cm) that remained at each time during the enzymatic treatment. Operating conditions identical to Figure 2.

Microscopic observations of the initial suspension revealed a large dispersion of non-spherical particles morphology and size (data not shown). Due to these heterogeneities, focus beam reflectance measurements (FBRM) and dynamic laser light diffraction (LLD) were employed to determine the number-weighted and volume-weighted particles distribution during enzymatic treatment with Rovabio® and xylanase C. With LLD method, one must keep in mind that the accuracy of the measurements is based on the criteria that the particles must be spherical giving rise to a diameter of equivalent sphere, d_se_ and that the refractive index of these particles in suspension are assumed to be known and constant. In addition, particle density is assumed to be constant whatsoever the size generated along the enzymatic hydrolysis. Since in our case, the particles were in majority non-spheric and heterogeneous, volume-weighted distribution function (*E*_*v*_) of sphere equivalent diameter (d_se_) was determined and multiplied by dry matter, in order to take into account the loss of insoluble fraction material throughout the enzymatic treatment. As shown in Figure 3, three classes of PSD could be identified relative abundance of which changed along the treatment. Class 3 ranging from 300 to over 1,100 μm was dominant (85% vol/vol) before any enzymatic treatment and exhibited a 30% drop in <30 min after the addition of Rovabio® (Figure 3A), whereas it required 6 h in the presence of xylanase to reach the same drop (Figure 3B). Moreover, the decrease of this class was not counterbalanced by an increase in abundance of class 2 (particle size between 50 and 300 μm) and class 1 (particle size <50 μm), which was likely due to the solubilization of particles.

**Figure 4:**
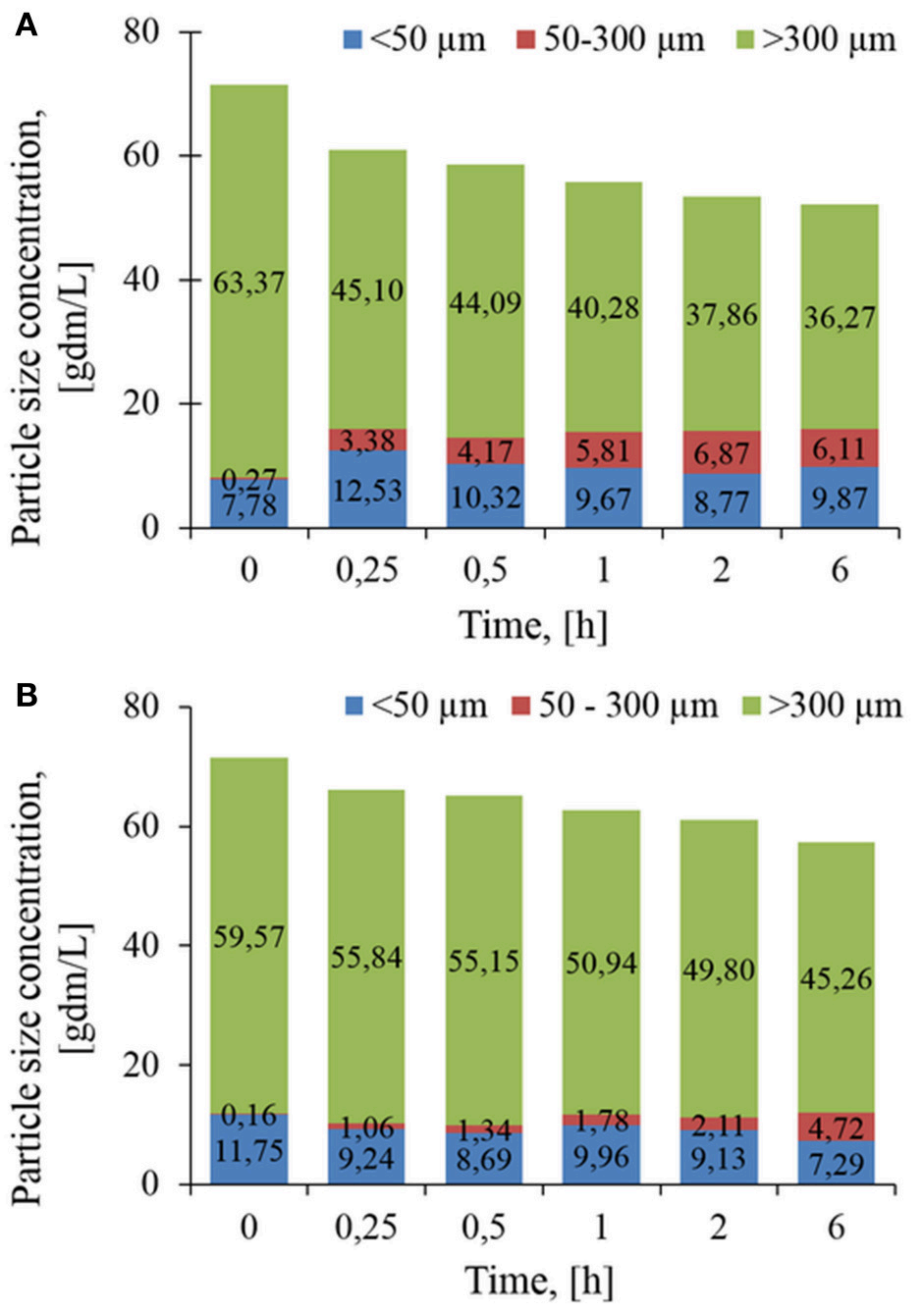
Evolution in the abundance of population class as determined by their chord length, lc during the treatment of the wheat bran suspension by Rovabio® **(A)** and xylanase C **(B)**. Chord length distribution, En(lc) was mulitplied by the total number of counted particles per sec (Nc). Operating conditions identical to Figure 2.

To better quantify the evolution of PSD, volumetric distributions were converted into concentration. As reported in Figure 4, the loss of 27.1 g/L of class 3 particles after 6 h of treatment with Rovabio®, was accompanied by an increase of 7.9 g/L of class 1 and 2, indicating that the remaining 19.2 g/L had been solubilized. In addition, this analysis revealed that it was mainly class 2 that became enriched through the treatment with Rovabio® as the abundance of this class raised from 0.27 to 6.1 g/L after 6 h of treatment whereas class 1 increased by only 25% (from 7.78 to 9.87 g/L). As for Rovabio®, class 3 was the main target of xylanase C, the abundance of which decreased by about 14.3 g/L over the 6 h of enzyme treatment. However, contrary to Rovabio®, class 1 decreased by 38% (from 11.75 to 7.29 g/L) whereas class 2 only showed a slight increase from 0.16 to 4.72 g/L. These data pointed out again a difference between these two treatments which can be explained by the presence of many hydrolytic enzymes in the cocktail that exert a synergistic action on wheat bran deconstruction.

**Figure 5:**
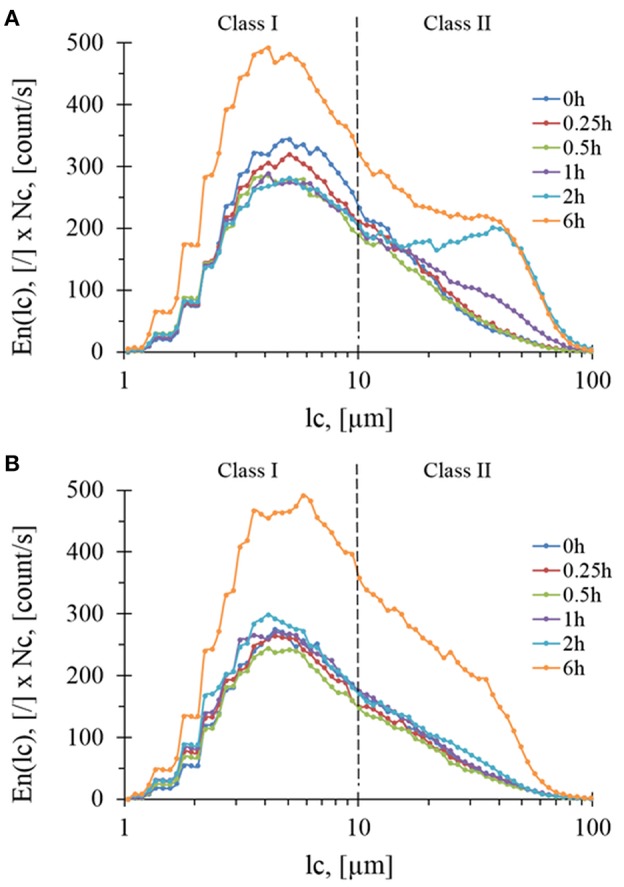
Evolution of particles concentration per class during enzymatic treatment of the destarched wheat bran by Rovabio® **(A)** and xylanase C **(B)** for different hydrolysis times. Operating conditions identical to Figure 2. Note that the concentrations is g of particles per liter of suspension.

To complete volume-weighted PSD that makes emphasis on coarse particles (dse > 100 μm), the FBRM technique was employed to investigate the evolution of fine particles during the treatment. In this case, the number-weighted distribution (En) as a function of chord length (lc) was considered. By multiplying *E*_*n*_(*l*_*c*_) by the total number of particles counted *per sec* (*Nc*), loss of insoluble matter by the solubilization was taking into account and hence monitor the evolution of chord length, *lc*. Accordingly, two subpopulations termed class I with 1 < lc < 10 μm (centered around 4.5 μm) and class II with 10 < lc < 100 μm (centered around 45 μm) were identified (Figure 5) whose abundances increased an reached after 6 h of treatment same level with both Rovabio® and xylanase C. However, it was noticed a clear difference between the two treatments, as class II increased earlier (at 1 h of treatment) in upon incubation with the industrial cocktail (see Figure S2).

### Mass Balance and Biochemical Analyses of the Deconstruction of Destarched Wheat Bran

**Figure 6:**
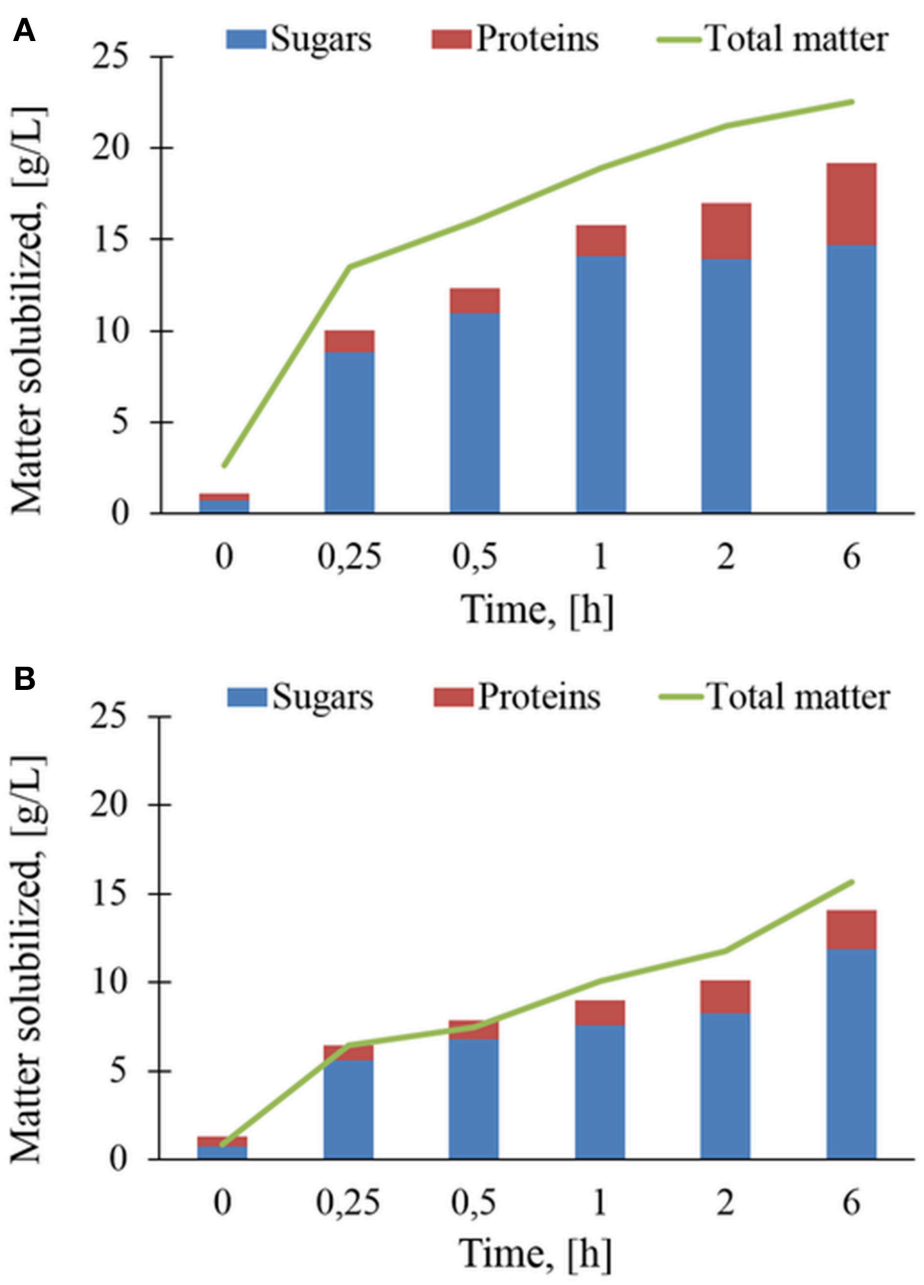
Sugars, proteins, and total matter (g/L) solubilized during enzymatic treatment of the destarched wheat bran by Rovabio® **(A)** and xylanase C **(B)**. Operating conditions identical to Figure 2. Note that concentration is g of matter solubilized per liter of supernatant.

The *in-situ* analyses reported above clearly indicated that the enzymatic action of Rovabio® and xylanase C caused a release of soluble material in the supernatant from insoluble destarched wheat bran. As a first approach, solubilisation rate was determined as the total matter released in the supernatant during these enzymatic treatments. Figure 6 shows that the solubilization rate with Rovabio® was roughly 3 times faster than with xylanase C within the first 30 min. After this time, the kinetic of solubilization suddenly dropped to continue at a very slow pace until the end. Since the solubilization mainly corresponded to release of sugars from NSPs hydrolysis and proteins, we quantified these two compounds in the supernatant. It can be shown in Figure 6 that the sum of solubilized sugars and proteins was between 12 and 15% below the total matter solubilized by Rovabio®, whereas this difference was less marked with xylanase C (see also Tables S1, S2 for the proportion of sugars and proteins in the solubilized fraction). This result suggested that the deconstruction with the industrial cocktail led to the release of other yet uncharacterized components in the supernatant. It can be also pointed out that the release of sugars stopped after 1 h in the presence of Rovabio® whereas it continued at a slow rate with xylanase C. Furthermore, the proportion of proteins in the solubilized matter increased more significantly upon treatment with the industrial cocktail than with xylanase, which could be attribute to proteases in Rovabio® (Guais et al., 2008).

Overall, the total matter solubilized after 6 h of treatment was 19.2 g/L with the industrial cocktail (notice that about 3,3 g/L was solubilized at time 0 independently to enzymatic hydrolysis) and 14.3 g/L with xylanase C, which corresponded to 28 and 19,5 % of the initial total destarched wheat bran, respectively (Tables S1, S2). It is worth to note that this moderate solubilization was not due to an inhibition of Rovabio® by products released during the treatment. Evidence for this assertion was notably to find that addition of a fresh solution of Rovabio® to destarched wheat bran that has been resuspended in the medium solution of a 6-h treated wheat bran with the same cocktail resulted in a same solubilization as with a fresh medium. This result is thus in accordance with the finding that addition of large excess of Rovabio® to a 6-h treated wheat bran did not further decreased the viscosity.

**Figure 7:**
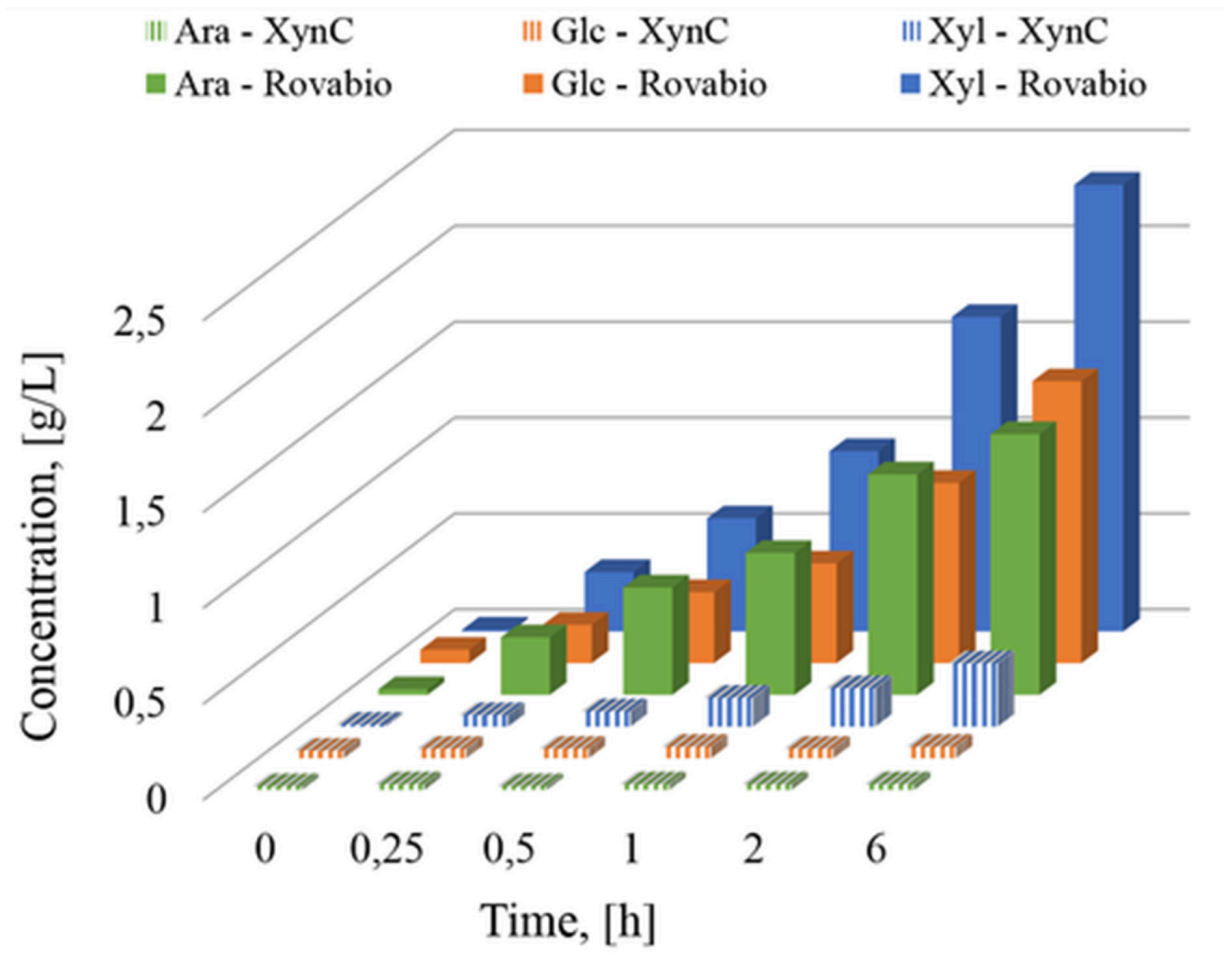
Determination of monosaccharides solubilized (g/L) during treatment of destarched wheat bran by Rovabio® or by XynC at equivalent activity to 400 U_xylanase visco_/gdm.

**Figure 8:**
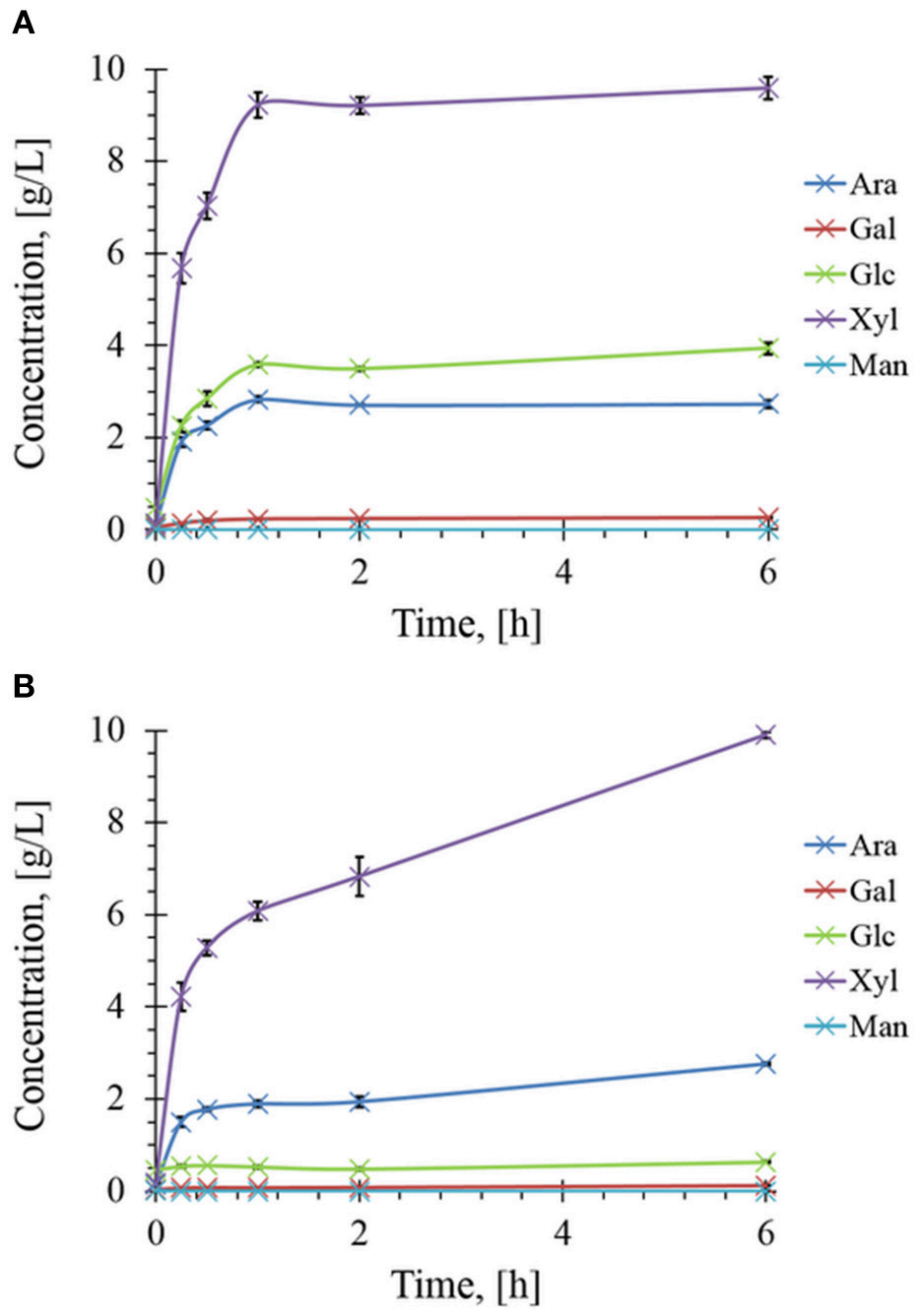
Kinetics of soluble sugars released during enzymatic treatment of the destarched wheat bran Rovabio® **(A)** and xylanase C **(B)**. Operating conditions identical to Figure 2. The soluble sugars were expressed as equivalent of monosaccharides glucose (Glc), xylose (Xyl), arabinose (Ara), mannose (Man), and galactose (Gal) after their chemical acid hydrolysis as described in Material and Methods.

The presence of different and complementary glycoside hydrolases activities in Rovabio® (Guais et al., 2008; François and Guais, 2011) can account for a direct release of xylose, glucose and arabinose in the supernatant of destarched wheat bran suspension (Figure 7). In contrast, only xylose was found in the supernatant of the destarched wheat bran treated with xylanase C, and its release was 6 times lower than during the treatment with the industrial cocktail. In addition, it is expected that these supernatants also contained soluble oligosaccharides released during these enzymatic treatments, which could be evaluated by acid-hydrolysis of the supernatant. The amount of monosaccharides (i.e., xylose, glucose, arabinose, or mannose) released from these oligosaccharides could be determined by subtracting the monosaccharide obtained after acid treatment from those directly measured in the supernatant before hydrolysis. Accordingly, oligosaccharides composed with a higher proportion of xylose, followed by glucose and arabinose were first released and plateaued at 1 h after addition of Rovabio® to the suspension (Figure 8). As expected, the production of oligosaccharides was faster and greater at the start of the treatment and was followed by their hydrolysis into the corresponding monosaccharides (Figure S3). Release of oligosaccharides was also observed during treatment of destarched wheat bran with xylanase C (Figure 8), except that only xylose and arabinose were identified as monosaccharides in the solubilized oligosaccharides. Interestingly, after a rapid phase of production, which lasted <1 h, oligosaccharides continued to be released from the insoluble material at a very slow rate during incubation with xylanase C to reach after 6 h, a similar proportion of xylose and arabinose as after 1 h treatment with Rovabio® (Figure 8).

## Discussion

The purpose of this study was to investigate the mechanism by which Rovabio®, an industrial enzyme cocktail currently used in animal nutrition (Guais et al., 2008), degrades NPSs that are abundantly present in wheat bran. A major result of this study was to show that Rovabio® exerts its deconstructive activity on wheat bran by two concurrent processes, namely a particles fragmentation and a solubilization. Such a concurrent event is expected to occur with complex polysaccharides presenting morpho-granulometry heterogeneities but it has often escape to the analysis likely because enzymatic deconstruction of biomass has rarely been investigated by combining physical and biochemical methods (Nguyen et al., 2015). This concurrent activity of Rovabio® resulted in a 70% drop of viscosity in <2 h, which can be mainly attributed to particles fragmentation (mainly largest ones), according to previous works showing a correlation of suspension viscosity and particles size with a dominant contribution of the bigger particles (Dasari and Eric Berson, 2007; Geddes et al., 2010). Nonetheless, the solubilization process could contribute to this drop of viscosity by reducing their size and thus affecting the surface properties of the particles.

Another significant result of this study was to confirm the synergistic effects of enzymes mixture in the degradation of complex polysaccharides (Van Dyk and Pletschke, 2012), as deconstruction of destarched wheat bran by xylanase C was slower and weaker than with the industrial cocktail. In addition, the overtaking of the viscosity after its 20% drop may be explained by the fact that xylanase C could only open fibers structure without strict fragmentation or could only unspool surface fibers leading to greater particle interactions and higher yield stress. The synergistic effects obtained with the industrial cocktail was also illustrated by the solubilization of other matters such as proteins and uncharacterized components. In addition, the weak fragmentation of particles upon incubation with xylanase C raised the question whether this could be due to a mechanical disruption of particles by the mixing system that results from the weakening of the particles structure due to the hydrolytic activity of this enzyme. However, we noticed that as much as arabinoxylans were solubilized after 6 h of treatment by xylanase C alone as by Rovabio® after 1 h. This result may suggest that the slower hydrolysis of arabinoxylans by xylanase C alone is due to the lack of some enzymatic components that contribute to a better accessibility of xylanase to its substrate.

A third important result of this study was to find that the fragmentation of particles could continue albeit at a slow rate independently to any sugar solubilization. This finding has never been reported before likely because at variance to this study, investigation of enzymatic deconstruction of biomass such as lignocellulose is often preceded by mild chemical pretreatments whose effects are to destroy structural characteristics of the recalcitrant biomass allowing easy accessibility of the fibers to enzymatic attack (Alvira et al., 2010). While we cannot exclude at this moment that this fragmentation event is the result of particle dismantling caused by the mixing system in spite of soft mixing condition, we can also formulate the hypothesis that this fragmentation activity is attributed to the action of “non-hydrolytic” accessory proteins. Example of such proteins are the swollenin, which can open up and disaggregate the less-ordered regions of lignocellulosic substrate (Cosgrove, 2017). However, the content of swollenin protein in Rovabio® is very low (O. Guais, personal communication), although the genome of the filamentous fungi used to produce this cocktail contains two genes encoding swollenins (François and Guais, 2011). Alternatively, or complementary, the non-catalytic carbohydrate binding modules (CBM) that are present at the N or C-terminal of sequence of several glycoside hydrolases (Guillen et al., 2010) could also contribute with swollenins to particles fragmentation, as it was reported for deconstruction of lignocellulose (Gourlay et al., 2012, 2015).

In spite of a rapid and effective action of Rovabio®, <30% of destarched wheat bran was solubilized leading to an insoluble fraction mostly enriched of particles with size <300 μm. The insoluble fraction still contained a large amount of NSPs that are apparently no longer accessible to the industrial cocktail. As this failure to completely hydrolyze NSPs was not caused by an inhibition of hydrolytic activities of this cocktail, this finding raised the question whether this largely incomplete hydrolysis of NSPs is due either to missing deconstructive/destabilizing activities in the cocktail and/or to physical inaccessibility of the hydrolytic enzymes to the substrate. In regards to the first possibility, it has been shown that unproductive or retardation of biomass degradation can be due to the presence of lignin (Himmel et al., 2007) and/or to tight adherence through hydrogen bonds of xylans to the surface of cellulose microfibrils (Simmons et al., 2016). Enzymes of the lytic polysaccharide monoxygenases (LPMOs) family have been shown to overcome these negative effects on degradation through their oxidative activity (Vaaje-Kolstad et al., 2010; Couturier et al., 2018). Since lignin content in wheat bran is very low (<5%), the possibility that LPMOs would increase degradation of wheat bran should be tested because Rovabio® does not contain any LPMOs. On the other hand, increasing enzymatic accessibility of particles by means of a mechanical pulverization that would increase the surface/volume should be tested as this process would mimic what is occurring in poultry digestive system (Svihus, 2014).

## Conclusions

An instrumented bioreactor allowing combined *in-situ* physical and *ex-situ* biochemical analyses was devised to investigate the mechanism by which non-starch and non-digestive polysaccharides abundantly present in wheat bran are deconstructed and degraded by Rovabio®, an industrial enzymatic cocktail commonly used as feed additive in animal nutrition. Our results unraveled that the deconstruction of this complex and insoluble matter implies a concomitant particles fragmentation and solubilization that requires the synergistic action of various glycoside hydrolases and auxiliary proteins. However, in spite of the numerous hydrolytic enzymes in Rovabio®, the solubilization did not exceed 30% of wheat bran dry mass, raising the question whether the deconstruction is restricted by lack of deconstructive/destabilizing enzymes and/or by physical inaccessibility to the residual fraction. Finally, this multiscale approach that encompasses a dynamic analysis of wheat bran deconstruction could be easily extended to study other raw and complex plant biomass degradation as well as to the design a customized enzyme cocktail for a given substrate.

## Data Availability

All datasets generated for this study are included in the manuscript and/or the Supplementary Files.

## Author Contributions

JF and LF conceived the study. MD refined the project plan, established the protocols, accordingly, and carried out the bioreactors under the supervision of XC and LF. MD performed the analytical experiments, with the guidance of OG and VN-R. JF, MD, and LF wrote the manuscript. All authors made comments on the manuscript and approved the final version.

### Conflict of Interest Statement

VN-R and OG have competing financial interest as they are employees of the ADISSEO Company that provides support to this study. The remaining authors declare that the research was conducted in the absence of any commercial or financial relationships that could be construed as a potential conflict of interest.

## Supplementary Material

Figure S1 Proteomic content of the Rovabio® enzyme coktail used in this study. The proteomic analysis of a sample of liquid Rovabio® was realized by a shot gun procedure as described in Guais et al. (2008). The different proteins was classified according to Cazymes classification (http://www.cazy.org/) and reported as % of the total proteins content.

Figure S2 Evolution of subpopulation proportions per class and the number of count per second (Nc) during destarched wheat bran treatment with Rovabio **(A)** and xylanase C **(B)**. Operating conditions identical to Figure 2.

Figure S3 Proportion of monosaccharide in the total soluble sugars released (in opposition to oligosaccharide forms) during enzymatic treatment of destarched wheat bran by Rovabio. Operating conditions identical to Figure 2.

Table S1 Sugars, proteins and total matter (%w/w initial dry matter) solubilized during enzymatic treatment of the destarched wheat bran by Rovabio Advance.

Table S2 Sugars, proteins and total matter (%w/w initial dry matter) solubilized during enzymatic treatment of the destarched wheat bran by xylanase C (B). Operating conditions identical to Figure 2.

## Abbreviations

CBM: carbo-binding module
CLD: chord length distribution
Cm: dry matter concentration (g/L)
d_*se*_: Diameter of sphere equivalent (μm)
FBRM: Focused Beam Reflectance Measurement
Kp: Power constant (/)
Ks: Metzner-Otto coefficient (/)
l_*c*_: Chord length (μm)
LLD: Laser Light Diffraction
*Nc*: number of chord counted (/)
*Np*: mixing power number (/)
*N*p_0_: mixing power number for turbulent flow (/)
NSP: non-starch polysaccharides
NSPases: non-starch polysaccharides hydrolases
PSD: Particle Size Distribution
*Re*: mixing Reynolds number (/)
RPM: rotation per minute (1/min).
