# Supplementary figures and images for: Combined in situ Physical and ex-situ Biochemical Approaches to Investigate in vitro Deconstruction of Destarched Wheat Bran by Enzymes Cocktail Used in Animal Nutrition
Source: Front Bioeng Biotechnol. 2019 Jun 26;7:158. doi: 10.3389/fbioe.2019.00158 (PMC6607472; doi:10.3389/fbioe.2019.00158)

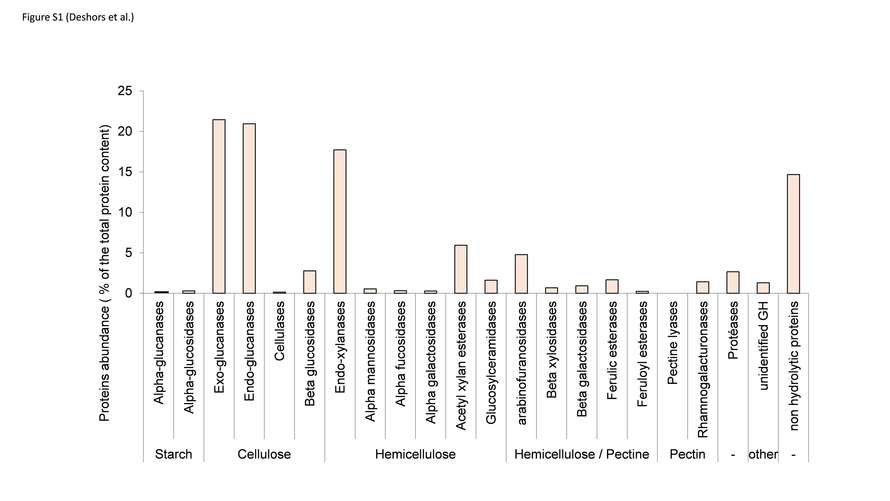

Supplement: Figure S1 — Proteomic content of the Rovabio® enzyme coktail used in this study. The proteomic analysis of a sample of liquid Rovabio® was realized by a shot gun procedure as described in Guais et al. (2008). The different proteins was classified according to Cazymes classification (http://www.cazy.org/) and reported as % of the total proteins content. [file Image_1.tif]

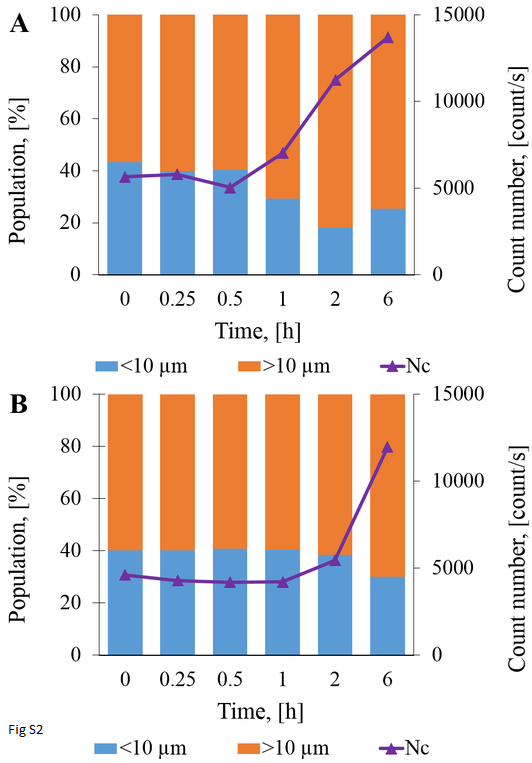

Supplement: Figure S2 — Evolution of subpopulation proportions per class and the number of count per second (Nc) during destarched wheat bran treatment with Rovabio (A) and xylanase C (B). Operating conditions identical to Figure 2. [file Image_2.tif]

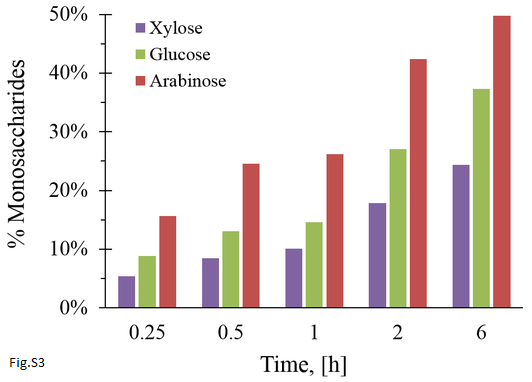

Supplement: Figure S3 — Proportion of monosaccharide in the total soluble sugars released (in opposition to oligosaccharide forms) during enzymatic treatment of destarched wheat bran by Rovabio. Operating conditions identical to Figure 2. [file Image_3.tif]
