# Supplementary material for: Combined in situ Physical and ex-situ Biochemical Approaches to Investigate in vitro Deconstruction of Destarched Wheat Bran by Enzymes Cocktail Used in Animal Nutrition
Source: Front Bioeng Biotechnol. 2019 Jun 26;7:158. doi: 10.3389/fbioe.2019.00158 (PMC6607472; doi:10.3389/fbioe.2019.00158)
Supplement: Table S1 — Sugars, proteins and total matter (%w/w initial dry matter) solubilized during enzymatic treatment of the destarched wheat bran by Rovabio Advance. [file Table_1.pdf]

Table S1: Sugars, proteins and total matter (%$_{w/w}$ initial dry matter) solubilized during enzymatic treatment of the destarched wheat bran by Rovabio Advance.

	0 h[$]	*0.25 h*	*0.5 h*	*1 h*	*2 h*	*6 h*
Solubilized matter	0	14.68	18.06	21.98	25.11	27.85
Sugars	0	11.00	14.82	18.09	17.83	18.88
Proteins	0	1.63	1.37	1.75	3.63	5.58
Δ*	0	2.05	1.87	2.14	3.65	3.49

*Δ is the difference between the solubilized matter and the sum of sugars + proteins in the soluble fraction. The data are from Fig.2.

[$] As there was some solubilization during the period of homogenisation in the bioreactor before addition of the enzyme, the values measured at time 0h for sugars, proteins and total soluble matter were
