# Supplementary material for: Combined in situ Physical and ex-situ Biochemical Approaches to Investigate in vitro Deconstruction of Destarched Wheat Bran by Enzymes Cocktail Used in Animal Nutrition
Source: Front Bioeng Biotechnol. 2019 Jun 26;7:158. doi: 10.3389/fbioe.2019.00158 (PMC6607472; doi:10.3389/fbioe.2019.00158)
Supplement: Table S2 — Sugars, proteins and total matter (%w/w initial dry matter) solubilized during enzymatic treatment of the destarched wheat bran by xylanase C (B). Operating conditions identical to Figure 2. [file Table_2.pdf]

Table S2: Sugars, proteins and total matter ($\%_{w/w}$ initial dry matter) solubilized during enzymatic treatment of the destarched wheat bran by xylanase C (B). Operating conditions identical to Fig.2.

	0 h$	*0.25 h*	*0.5 h*	*1 h*	*2 h*	*6 h*
Solubilized matter	0	7.7	9.13	12.72	15.08	19.47
Sugars	0	6.8	8.4	9.74	11.42	15.89
Proteins	0	0.4	0.67	1.15	1.69	2.23
Δ	0	0.50	0.07	1.83	1.97	1.35

*Δ is the difference between the solubilized matter and the sum of sugars + proteins in the soluble fraction. The data are from Fig.2.

$As there was some solubilization during the period of homogenisation in the bioreactor before addition of the enzyme, the values measured at time 0h for sugars, proteins and total soluble matter were subtracted from those at different time after the addition of the xylanase
